# Impact of Housing on Burn Injury Patterns and Outcomes: A Retrospective Cohort Study at a Canadian Burn Center

**DOI:** 10.1093/jbcr/iraf107

**Published:** 2025-06-09

**Authors:** Justin J Lee, Izza Sattar, Sean Harrop, Trent Schimmel, Shawn X Dodd, Sharada Manchikanti, Joshua N Wong, Alexis Armour

**Affiliations:** Division of Plastic and Reconstructive Surgery, Department of Surgery, University of Alberta, Edmonton, Alberta, Canada; Faculty of Medicine and Dentistry, University of Alberta, Edmonton, Alberta, Canada; Faculty of Medicine and Dentistry, University of Alberta, Edmonton, Alberta, Canada; Division of Plastic and Reconstructive Surgery, Department of Surgery, University of Alberta, Edmonton, Alberta, Canada; Division of Plastic and Reconstructive Surgery, Department of Surgery, University of Alberta, Edmonton, Alberta, Canada; Division of Plastic and Reconstructive Surgery, Department of Surgery, University of Alberta, Edmonton, Alberta, Canada; Division of Plastic and Reconstructive Surgery, Department of Surgery, University of Alberta, Edmonton, Alberta, Canada; Division of Plastic and Reconstructive Surgery, Department of Surgery, University of Alberta, Edmonton, Alberta, Canada

**Keywords:** social determinants of health, housing, no fixed address, frostbite, discharge against medical advice, burn injury, thermal injury

## Abstract

Houselessness is an important social determinant of health, which may predispose to poor health and difficulty in recovering from new health issues. We examined the demographic variables and clinical outcomes of housed and unhoused patients experiencing thermal injuries. A retrospective chart review was performed on all patients admitted to or followed by the burn service from January 2022 to June 2024. There were 571 new thermal injuries requiring admission at our institution, including 414 patients with housing and 157 unhoused patients. Frostbite accounted for 35% of admissions among unhoused patients, which was significantly greater than the 10% of housed patient admissions for frostbite injuries (*P* < .0001), where thermal burns accounted for the majority of injuries requiring admission. Substance use was significantly higher in the unhoused population (*P* < .0001). Unhoused patients on the burn service were 10-fold more likely to leave against medical advice, compared with housed patients (*P* < .00001). Interestingly, the mean length of stay was not significantly different between the housed and unhoused inpatients; however, it was significantly different with the exclusion of patients who left against medical advice (18.21 *±* 30.84 days vs 24.70 *±* 26.30 days; *P* = .03). The houseless population in Canada experiences unique challenges due to the extreme cold, while their inpatient-care providers are challenged by a resource-constrained system. These results suggest the need for additional support for thermal injury prevention and substance use disorder treatment among the unhoused population.

## INTRODUCTION

Houselessness is an important social determinant of health, which may predispose to poor health and difficulty in recovering from new health issues.^1^^,^^2^ Numerous measures of health outcomes have been investigated in the context of houselessness including higher prevalence of mental health disorders, cardiovascular disease, traumatic brain injury, infectious diseases, burns, hypothermia, and frostbite, as well as greater use of emergency departments and longer average length of stay (LOS) in hospital.^3-9^ Studies from the United States investigating burn injuries have identified that individuals experiencing houselessness are more likely to encounter flame burns, self-inflicted or assault-related burns, and more full-thickness injuries than the housed population.^3-9^ Frostbite in the houseless population has similar trends with higher incidence of frostbite, more severe injury, and higher likelihood of amputation,^3^ as well as higher incidence of hypothermia.^7^^,^^10^ The demographics and outcomes of patients with burn injuries have been studied in urban centers in the United States^4^^,^^5^^,^^11-13^; however, there is a paucity of data in a Canadian context to inform Canadian healthcare and housing policies.^14^

Between 2018 and 2022, the estimated number of people experiencing houselessness in Canada increased by 20% with the proportion of unsheltered individuals increasing from 14% to 23%.^15^^,^^16^ The 2022 report also found that 33% of people experiencing houselessness in Western Canada were unsheltered, as opposed to 17% in Central Canada and 19% in Eastern Canada. Two studies have investigated frostbite and hypothermia in Central Canada,^7^^,^^10^ but little is known about the demographics and outcomes of patients with burn and frostbite injuries in Western Canada.

Edmonton is an urban center in Western Canada with a population over 1.1 million^17^ and a growing houseless population.^18^^,^^19^ The houseless population in December 2024 was estimated to be 4963 with 24% (1239) being unsheltered.^19^ These values have more than doubled since the 2018 point-in-time count that found 2149 people experiencing houselessness with 18% (392) being unsheltered.^19^ The increased number of unsheltered individuals in Edmonton is of growing concern as there has been an overall reduction in shelter beds^20^ and increased encampment clearing since the pandemic.^21^ Inadequate protection from the harsh winter climate increases dependence on unsafe heating sources to cook and stay warm, thus increasing the risk for both frostbite and burns.^11^^,^^20^^,^^21^

This study aims to characterize demographics and outcomes of patients experiencing houselessness who sustained thermal burn or frostbite injury and compare them with a cohort of thermally injured patients with stable housing. We hypothesize that individuals with unstable housing will have a higher complication rate, injury severity, and increased LOS with thermal and frostbite injuries than patients with stable housing.

## METHODS

After institutional health research ethics was obtained (Pro00139975), a retrospective cohort study was conducted using data from electronic medical records on Epic (Verona, USA) at the University of Alberta Hospital, which is an American Burn Association (ABA)-verified burn center. Data were obtained for patients admitted to or consulted by the burn service from January 1, 2022 to June 30, 2024. Exclusion criteria consisted of any patients admitted to the burn service for any nonburn and nonfrostbite injury, such as patients admitted for necrotizing fasciitis, Stevens-Johnson syndrome and toxic epidermal necrolysis. Patients were included as a readmission if they were readmitted to the burn unit during the time period of the study for complications related to the initial presenting injury including revision surgery, infection, and wound dehiscence.

A chart review was conducted to determine demographics, housing status, admission diagnosis, injury characteristics, comorbid diagnoses, complications, length of hospital stay, and disposition. A patient was considered to be unhoused if they had no fixed address at the time of admission. The primary objective was to characterize demographics and outcomes of inpatients with unstable housing who sustained burn injury from thermal, frostbite, chemical, or electrical mechanisms, and to compare them with patients having stable housing. Secondary objectives included complications, comorbidities, disposition status, and LOS between the 2 groups. Complications were defined as any unfavorable outcome arising after admission to the burn service, including substance overdose from own supply, infection, cardiovascular complications, surgical complications, and noncompliance with treatment protocols.

Comparative analyses between groups were performed using SPSS statistical software (Version 28.0; IBM Corp., Armonk, NY; 2021). Statistical analyses were conducted using one-way analyses of variance, Student’s t-tests, chi-squared tests, and/or their equivalent for nonparametric comparisons. *P*-value was set at .05 for statistical significance with a power (1 − β) of 0.8.

## RESULTS

A total of 571 consecutive adult patients were admitted to the University of Alberta Hospital for burn injuries; 414 (72%) were housed and 157 (28%) were unhoused. Table 1 outlines demographic data for the housed and unhoused patient cohorts. No significant differences were found between age and sex of the housed and unhoused population. Males made up the majority of both housed (73%) and unhoused (71%) cohorts.

**Table 1:** Demographic Information of Housed and Unhoused Patients

	Housed (*n* = 414)	Unhoused (*n* = 157)	*P*-value
Age (y), mean ± SD	40.7 ± 24.0	40.9 ± 13.0	.92
Sex, *n* (%)			
Male	304 (73.4)	112 (71.3)	.84
Female	110 (26.6)	45 (28.7)	.71

Table 2 describes the burn and frostbite injury characteristics between housed and unhoused cohorts. Comparing housed with unhoused cohorts, there was no significant difference in the mean burn size of total body surface area (TBSA; 10.1% vs 12.6%), but there was a significantly greater percentage of full-thickness vs partial-thickness burns among unhoused patients, compared with the housed group (58.8% vs 35.4%, *P* = .011). A significantly greater proportion of unhoused patients had frostbite injuries compared with housed patients (35.0% vs 10.6%, *P* < .001), although there was no significant difference in the grade of frostbite injury between housed and unhoused cohorts. Only housed patients presented with chemical burns (*n* = 13) and electrical burns (*n* = 11), which represented 3.1% and 2.7% of presenting injuries in the housed cohort. No chemical or electrical burn injuries were observed among the unhoused inpatients during the time period of this study.

**Table 2:** Injury Characteristics for Housed and Unhoused Patients with Burn Injuries

	Housed (*n* = 414)	Unhoused (*n* = 157)	*P*-value
Thermal, *n* (%)	345 (83.3)	102 (65)	.089
Depth, *n* (%)			
Superficial	13 (3.7)	1 (1)	1.000
Partial thickness	210 (60.9)	41 (40.2)	**.033**
Full thickness	122 (35.4)	60 (58.8)	**.011**
%TBSA, mean ± SD	10.14 ± 14.51	12.59 ± 18.23	.170
Frostbite, *n* (%)	44 (10.6)	55 (35)	**<.00001**
Grades 1 and 2, *n* (%)	22 (50)	30 (54.5)	.699
Grades 3 and 4, *n* (%)	22 (50)	25 (45.4)	.724
Chemical, *n* (%)	13 (3.1)	0 (0)	**.025**
Electrical, *n* (%)	11 (2.7)	0 (0)	**.042**

Abbreviation: *TBSA*, total body surface area. Boldface refers to statistical significance of *P* < .05.

Table 3 lists the comorbid conditions of the housed and unhoused cohorts at the time of admission to hospital. Unhoused patients were significantly more likely to have comorbid substance use disorder (SUD; 43% vs 7%, *P* < .001) and infections (8% vs 2%, *P =* .002) when compared with the housed patients. Conversely, housed patients had significantly more renal disease (5% vs 1%, *P* = .002) than the unhoused cohort. No significant differences were found with cardiac, hematological, respiratory, or trauma-related comorbidities between the 2 groups.

**Table 3:** Comorbid Conditions at the Time of Admission of Housed and Unhoused Patients

	Housed (*n* = 414)	Unhoused (*n* = 157)	*P*-value
Substance use, *n* (%)	28 (7)	68 (43)	**<.0001**
Renal disease, *n* (%)	20 (5)	1 (1)	**.017**
Respiratory, *n* (%)	20 (5)	4 (3)	.225
Trauma, *n* (%)	15 (4)	4 (3)	.522
Cardiac, *n* (%)	15 (4)	5 (3)	.799
Infection, *n* (%)	9 (2)	12 (8)	**.002**
Hematological, *n* (%)	4 (1)	1 (1)	.706

Boldface refers to statistical significance of *P* < .05.

Table 4 shows the clinical characteristics and disposition outcomes of burn injury patients. Unhoused patients had a significantly greater number of readmissions (0.7 vs 0.3, *P* < .001), and number of procedures (1.5 vs 1.2, *P* < .05) related to their initial injury. Housed patients had significantly more hospital complications (15.4% vs 9.2%, *P <* .05). There were significantly more unhoused patients who were discharged to a shelter (21.0% vs 0.5%, *P <* .001) and who left against medical advice (AMA; 33.3% vs 3.4%, *P* < .001). Housed patients were significantly more likely to be discharged home (68.0% vs 27.0%, *P* < .001) or be transferred to another facility (26.0% vs 16.0%, *P* < .05) than the unhoused patients. No difference was found in the fatality rate between the 2 cohorts.

**Table 4:** Clinical Characteristics and Disposition Status of Housed and Unhoused Patients with Burn Injuries

	Housed (*n* = 414)	Unhoused (*n* = 157)	*P-*value
Clinical characteristics			
No. of readmissions, mean ± SD	0.33 ± 0.71	0.68 ± 1.1	**<.0001**
No. of procedures, mean ± SD	1.18 ± 1.75	1.49 ± 1.89	**<.050**
Presence of hospital complications, *n* (%)	38 (9.2)	24 (15.3)	**.030**
Disposition status, *n* (%)			
Home	280 (68)	42 (27)	**<.00001**
Shelter	2 (0.5)	32 (21)	**<.00001**
Transfer	107 (26)	25 (16)	**.045**
AMA/AWOL	14 (3.4)	52 (33.3)	**<.00001**
Died	10 (2.4)	5 (3.2)	.613

Abbreviations: *AMA*, against medical advice; *AWOL*, absent without leave. Boldface refers to statistical significance of *P* < .05.

Table 5 shows the average LOS in hospital for each type of injury. No significant differences were found in the LOS for thermal, frostbite, chemical, or electrical injuries between the housed and unhoused cohorts. The combined LOS was significantly different when the patients who left AMA were excluded, showing the unhoused cohort stayed in hospital for significantly more days (24.7 vs 18.2, *P <* .05).

**Table 5:** Average Length of Stay of Housed and Unhoused Patients with Burn Injuries

	Housed, mean (days) ± SD	Unhoused, mean (days) ± SD	*P*-value
Combined	18.12 ± 30.58	21.56 ± 25.05	.210
Combined^*^	18.21 ± 30.84	24.70 ± 26.30	**.030**
Thermal	17.88 ± 31.96	20.23 ± 20.94	.810
Frost	22.69 ± 24.05	24.07 ± 31.46	.470
Chemical	9.08 ± 6.85	N/A	
Electrical	17.82 ± 26.34	N/A	

^*^Excludes patients who left AMA. Boldface refers to statistical significance of *P* < .05.

## DISCUSSION

Houselessness is a significant factor in almost one-third of the patients admitted to the University of Alberta burn service for thermal and frostbite injuries. There are only a handful of studies from the US that show houseless patients experience larger burns and longer hospital LOS.^2-4^ Speiser et al. reported that since the COVID-19 pandemic, unhoused patients were more likely to leave AMA.^2^ To our surprise, there are no studies that describe injury characteristics and outcomes in a resource-constrained Canadian universal healthcare system. Therefore, we conducted a retrospective cohort study assessing the demographics, injury characteristics, and outcomes of housed and unhoused patients with burn injuries admitted to or consulted by the burn service at a single tertiary-care Canadian institution. We observed that (1) the unhoused population had a significantly greater proportion of frostbite injuries than the housed population; (2) a significantly greater number of the unhoused population at the time of admission had an active SUD (withdrawal or medical management); (3) a significantly greater proportion of the unhoused population discharged themselves AMA; (4) the unhoused population had a greater readmission rate, as well as a greater number of procedures per patient; and (5) the LOS was not significantly different in the housed and unhoused populations, when patients who left AMA were included.

Despite the relatively colder climate, there is a relative paucity of literature illustrating the demographics and outcomes related to frostbite injuries in Canada.^5-8^ A retrospective study at a single tertiary-care center in the same urban community of this study demonstrated 79 frostbite injury cases over a 10-year period with 53% of cases with substance use at the time of admission.^5^ Another 12-year retrospective study conducted in an adjacent prairie province involving 3 tertiary hospitals, showed 125 frostbite injury cases with predisposing factors of alcohol and substance use and assessed the rates of amputation.^6^ Within 2.5-year span from January 2022 to June 2024, we observed 85 frostbite injury adult patients who required admission to our institution. Direct comparison with the above mentioned studies is challenging given the population growth since the 1990s to the 2020s and different communities these institutions serve. However, this begs the question whether there was a significant impact of the opioid epidemic, the 2008 housing crisis, the COVID-19 pandemic, as well as climate change on the rates of severe frostbite injuries. A retrospective descriptive analysis conducted from Toronto demonstrated that rates per 100 000 emergency visits related to frostbite injuries were significantly higher in the postpandemic period than the prepandemic period with houselessness being a significant secondary variable in the female population.^8^ Our data highlight the magnitude of the risk of severe frostbite to the unhoused population, as demonstrated by the higher proportion of frostbite vs burn injuries in this group. A dedicated study investigating the rate of frostbite injuries per capita from the pre- and postpandemic era should be considered to further demonstrate the impact of housing on frostbite injuries and discuss preventative strategies to reduce the number of these injuries in vulnerable populations.

Patients who leave AMA or are absent without leave (AWOL) may be at risk of readmission, as well as adverse health outcomes.^9-11^ Factors impacting discharges AMA have been postulated to be affected by patient and provider factors.^12^^,^^13^ Patient factors include sociodemographic characteristics, admitting diagnosis, previous admission history, and substance use.^14^^,^^15^ Our data showed that a significantly greater proportion of unhoused patients with burn injuries were discharged AMA/AWOL compared with the housed population, with significantly greater number of readmissions in the unhoused patients, reflecting the impact of housing as one of the patient factors contributing to early discharge AMA. In addition, we observed a greater proportion of SUD in our unhoused burn population. To further investigate, we combined the housed and unhoused populations, and a comparison of disposition status was performed in the SUD vs non-SUD groups. This showed a significantly greater proportion of individuals with SUD leaving AMA compared with the non-SUD group (Table S1). Discussions from various retrospective, prospective, and qualitative studies investigating the impact of substance use on discharge AMA suggest that early recognition and treatment of withdrawal symptoms, destigmatization of SUD, and building good physician–patient relationships may prevent the number of patients leaving AMA.^10^^,^^16-18^ A reason why we may see this in our unhoused burn population is that at the time of this study our institution lacked an Addiction Medicine Consultation Team (AMCT) model. This is a multidisciplinary care team that assesses and treats SUD in acute care management, preventative and long-term follow-up settings, which has previously been shown to improve perceived patient-care outcomes within our community.^19^ It is the hope that our data, supported by the abovementioned studies, highlight the importance of substance use management in preventing early discharge AMA.

Provider factors postulated to affect patients leaving AMA include hospital setting and structure, staffing patterns, admission and discharge policies, and physicians’ clinical style and experience.^12^^,^^13^ A retrospective cohort study conducted at an ABA-verified burn center in Los Angeles from 2016 to 2018 showed that housing status did not significantly affect the number of patients that were discharged AMA.^3^ A subsequent retrospective cohort study from this group assessed the disposition outcomes of unhoused patients with burn injuries admitted in the prepandemic (2015-2020) and postpandemic (2021-2023) eras showed that the patients admitted in the postpandemic era had greater rates of discharge AMA.^2^ This was thought to be secondary to a distrust of medical professionals in relation to the COVID-19 pandemic.^2^^,^^20^ It would be of interest to analyze prepandemic vs postpandemic analyses of discharge AMA in housed and unhoused patient populations at our center, while also performing exploratory research on provider factors that may help reduce the rates of discharge AMA in the unhoused patient population.

Increased LOS in the houseless population has been observed in previous studies.^21-24^ This is thought to be secondary to system issues including lack of affordable housing and/or safe discharge destination, insurance coverage, as well as severity of illnesses.^21-24^ A study conducted in Alberta, which includes the University of Alberta Hospital, showed that on average the unhoused population spent 1.62 more hours in the emergency department and 3.02 more days in the hospital.^25^ This was reflected in our study. With exclusion of patients who left AMA, there was a significantly longer mean LOS in the unhoused population vs the housed population among those who were admitted with burn injuries (Table S2). Delays in discharge resulting in increased LOS are certainly of fiscal interest in the Canadian universal healthcare model, where overall healthcare costs have a direct impact on the provincial economy. In Edmonton, adaptation of “specialist homeless discharge” programs (eg, Respite to Recovery Program)^26^ has proven to be a cost-effective alternative to traditional discharge methods. This involves transfer of unhoused patients, who no longer require acute care, to a nurse-led subacute facility where patients receive daily wound care, medication management, and disposition social work planning.^25^^,^^27^ However, these programs are constrained by capacity, with only 28 beds available for more than 4000 unhoused individuals in Edmonton. A cost-analysis study, as well as an exploratory data assessment of increased LOS in the unhoused burn population, is beyond the scope of this study, but is of interest in the future.

In this study, we report the largest sample size of unhoused patients with burn injuries in Canada, as the northernmost ABA-certified burn center in North America, providing unique insight into housing and thermal injuries in a universal healthcare system model. There are several limitations in this study that should be acknowledged. This study was conducted with admission data from a single tertiary-care center, which limits its generalizability to other locations across Canada. Although all thermal, chemical, and electrical burn injuries are directed to the University of Alberta Hospital, some frostbite injuries requiring admission may be treated at other care centers across the city. Therefore, this may be underrepresented in our data. In addition, the catchment area of University of Alberta Hospital includes the northern half of the province of Alberta, as well as adjacent provinces and territories. This may overrepresent the proportion of the unhoused population requiring admission for burn injuries. Hence, per capita assessment was not conducted in this study. Lastly, some housed patients may initially be admitted with permanent housing, but due to prolonged hospitalization and limited economic stability, may become unhoused by the end of their admission. Conversely, some unhoused patients may be discharged to a family member’s home or be arranged for housing through social work. In the latter case, we have observed longer LOS, despite being ready for discharge from the acute-care perspective.

## CONCLUSION

Caring for the unhoused population in the Canadian healthcare system presents unique challenges. With respect to unhoused patients requiring admission for treatment of burn injuries, there was an increased proportion of frostbite injuries, AMA disposition, and LOS, which showed a significant association with SUD in unhoused patients leaving AMA. This study provided an opportunity for our multidisciplinary burn-care team to reassess our SUD management practices, while imploring hospital administration and advisors to expand AMCT models such as the Addiction Recovery and Community Health team.^28^ It is the hope that future studies will involve multicenter data to compare demographic and clinical outcomes of unhoused thermally injured patients across the country, while assessing current methodologies in preventing burn injuries and reducing discharge AMA, readmissions, and LOS in the unhoused population. We hope to continue working with local homeless advocacy groups, health officials, and municipal, provincial, and federal governments to prevent burn injuries and improve outcomes among the unhoused population in all communities.

## Supplementary Material

JBCR_Supplementary_Tables_20250126_iraf107

## Notes

Author contributions: Justin J. Lee (Conceptualization, Data curation, Formal analysis, Investigation, Methodology, Project administration, Writing—original draft, Writing—review & editing [lead]), Izza Sattar (Conceptualization, Data curation [supporting]), Sean Harrop (Validation, Writing—original draft, Writing—review & editing [supporting]), Trent Schimmel (Conceptualization, Investigation, Writing—review & editing [supporting]), Shawn X. Dodd (Conceptualization, Data curation, Validation, Writing—review & editing [supporting]), Sharada Manchikanti (Data curation [lead], Project administration [supporting]), Joshua N. Wong (Conceptualization, Data curation [supporting], Resources, Supervision [equal], Writing—original draft, Writing—review & editing [supporting]), and Alexis Armour (Conceptualization, Data curation, Formal analysis [supporting], Supervision [lead], Writing—original draft [supporting], Writing—review & editing [supporting])

Funding: Funding for this research was provided by the Edmonton Firefighters’ Burn Treatment Society.
