# Supplementary material for: Impact of Housing on Burn Injury Patterns and Outcomes: A Retrospective Cohort Study at a Canadian Burn Center
Source: J Burn Care Res. 2025 Jun 9;46(6):1237–42. doi: 10.1093/jbcr/iraf107 (PMC12596692; doi:10.1093/jbcr/iraf107)
Supplement: JBCR_Supplementary_Tables_20250126_iraf107 [file jbcr_supplementary_tables_20250126_iraf107.docx]

**Supplementary Table 1.** Impact of substance use on disposition status in all burn patients admitted or consulted by the burn service from January 2022 to June 2024.

|  | No substance use | | | Substance use | | |  | |  |
| --- | --- | --- | --- | --- | --- | --- | --- | --- | --- |
| House status | N | % |  | N | % |  | | *P*-value | |
| Home | 289 | 61.1% |  | 33 | 34.4% |  | | **0.007** | |
| Shelter | 16 | 3.4% |  | 18 | 18.8% |  | | **0.0001** | |
| Transfer | 113 | 23.9% |  | 19 | 19.8% |  | | 0.489 | |
| AMA/AWOL | 42 | 8.9% |  | 24 | 25% |  | | **0.0001** | |
| Died | 13 | 2.7% |  | 2 | 2.1% |  | | 1.0000 | |

**Supplementary Table 2.** Impact of substance use on average length of stay in housed and unhoused burn patients.

|  | No substance use | | | Substance use | | |  |
| --- | --- | --- | --- | --- | --- | --- | --- |
| House status | N | Mean | SD | N | Mean | SD | *P*-value |
| Housed | 386 | 18.11 | *±* 31.37 | 28 | 18.32 | *±* 16.45 | 0.97 |
| Unhoused | 89 | 18.42 | *±* 17.06 | 68 | 25.03 | *±* 31.72 | 0.10 |
| Total | 475 | 18.17 | *±* 29.22 | 96 | 23.07 | *±* 28.21 | 0.13 |
| Housed* | 372 | 18.24 | *±* 31.72 | 27 | 18.74 | *±* 16.61 | 0.936 |
| Unhoused* | 59 | 21.66 | *±* 18.99 | 45 | 30.84 | *±* 34.46 | 0.0862 |
| Total* | 431 | 18.71 | *±* 30.3 | 72 | 26.31 | *±* 29.53 | **0.0487** |

*Excludes patients who left AMA
